# Colonic stenosis caused by infection of an intraperitoneal access port system: a rare complication of intraperitoneal chemotherapy for gastric cancer with peritoneal metastasis

**DOI:** 10.1186/1477-7819-12-177

**Published:** 2014-06-04

**Authors:** Jun Kinoshita, Sachio Fushida, Tomoya Tsukada, Katsunobu Oyama, Toshifumi Watanabe, Koichi Okamoto, Isamu Makino, Keishi Nakamura, Hironori Hayashi, Hisatoshi Nakagawara, Tomoharu Miyashita, Hidehiro Tajima, Hiroyuki Takamura, Itasu Ninomiya, Hirohisa Kitagawa, Takashi Fujimura, Tetsuo Ohta

**Affiliations:** 1 Department of Gastroenterologic Surgery, Division of Cancer Medicine, Graduate School of Medical Science, Kanazawa University, 13-1 Takaramachi, Kanazawa, 920-8641, Japan

**Keywords:** Intraperitoneal chemotherapy, Gastric cancer, Peritoneal metastasis, Port complication

## Abstract

**Background:**

Intraperitoneal (IP) chemotherapy is garnering attention as an effective treatment for gastric cancer with peritoneal metastasis. We report the case of a patient who developed colonic stenosis caused by infection of an IP access port system during IP chemotherapy. It was difficult to differentiate whether the extrinsic colonic stenosis arose from a catheter infection or peritoneal metastasis of the gastric cancer.

**Case presentation:**

A 66-year-old Japanese man underwent total gastrectomy for gastric cancer. Because the intraoperative findings revealed peritoneal metastasis, a port system was implanted for subsequent IP chemotherapy. Two months after initiation of chemotherapy, he complained of vomiting and abdominal pain. A computed tomography scan revealed marked thickening of the sigmoid colon wall adjacent to the catheter of the IP access port system. A barium enema demonstrated extrinsic irregular stenosis of the sigmoid colon. Although it was difficult to distinguish whether infection or peritoneal metastasis had caused the colonic stenosis, we removed the port system to obtain a therapeutic diagnosis. Coagulase-negative staphylococci were detected by catheter culture. The wall thickening and stenosis of the sigmoid colon completely resolved after removal of the port system.

**Conclusions:**

We report the case of a rare complication in association with an IP access port system. Infection of the port system should be considered as a differential diagnosis when colonic stenosis adjacent to the catheter is observed during IP chemotherapy.

## Background

Gastric cancer is a major cause of cancer death worldwide. Recent advances in systemic chemotherapy regimens have shown encouraging tumor response rates and increased survival in patients with unresectable or metastatic gastric cancer [1]. However, treatment outcomes for patients with peritoneal metastasis, which is the most frequent metastatic pattern of recurrence, have not improved sufficiently [2].

Intraperitoneal (IP) chemotherapy is garnering attention as an effective treatment for peritoneal metastasis because of the theoretical advantage of higher local concentrations, prolonged tumor exposure, and reduced systemic toxicity [3,4]. IP chemotherapy was shown to prolong survival in a phase III study of ovarian cancer with peritoneal metastasis and has been approved as a recommended regimen by the National Cancer Institute in the United States [5]. IP chemotherapy has also been shown to be a promising treatment option for gastric cancer [6-9]. A multicenter randomized clinical trial is now ongoing to generate evidence regarding the effects of IP chemotherapy on gastric cancer with peritoneal metastasis.

A subcutaneous port and catheter system has been developed and is now the most common route through which chemotherapeutic agents are administered into the peritoneal cavity. The main advantages of subcutaneous systems are the low rate of port-related infections and the ease of drug administration into the peritoneal cavity [10]. However, complications associated with subcutaneous port systems have been reported in patients with ovarian cancer and gastric cancer [10-19].

We herein describe a patient who developed colonic stenosis caused by the infection of an IP access port system during IP chemotherapy for gastric cancer with peritoneal metastasis. It was difficult to differentiate whether the extrinsic colonic stenosis arose from a catheter infection or the peritoneal metastasis.

## Case presentation

**Figure 1:**
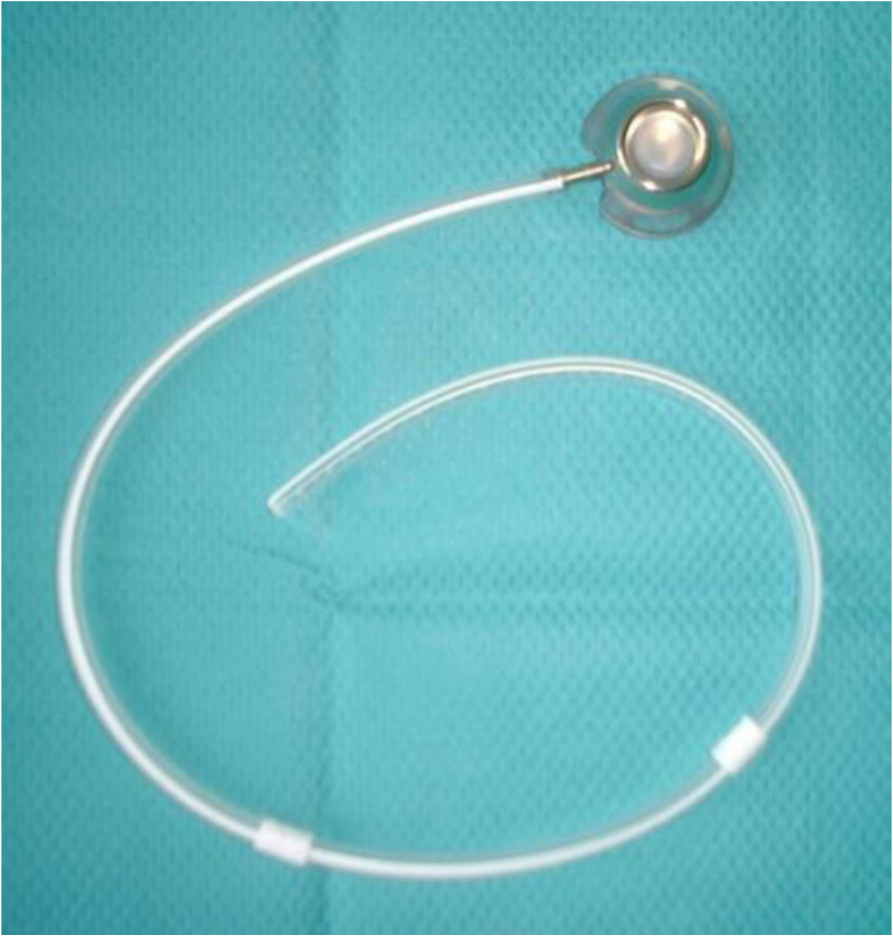
**Intraperitoneal access port system.** The intraperitoneal access port system comprised of a titanium port with a bottom diameter of 31.47 mm, a height of 14.5 mm, and a 14.3-Fr single-lumen silicone catheter.

A 66-year-old Japanese male patient was discovered to have anemia (hemoglobin level, 6.1 g/dL) at a medical checkup. He underwent upper gastrointestinal endoscopy, which revealed Borrmann’s type IV gastric cancer, and the biopsy findings resulted in a diagnosis of moderately differentiated adenocarcinoma. He then consulted with our hospital for surgical treatment.A detailed examination revealed T3N1M0 (stage IIB) cancer according to the Union for International Cancer Control tumor, node, and metastasis classification. Although a laparotomy was performed for curative resection, serosal invasion of the primary tumor and multiple peritoneal metastases in the peritoneal cavity were discovered intraoperatively. Peritoneal lavage cytology was also positive according to the Japanese classification of gastric carcinoma. We performed a total gastrectomy for cytoreduction and implanted an IP access port system (Bardport-Ti; CR Bard Inc., New Jersey, United States) comprising of a titanium port with a bottom diameter of 31.47 mm, a height of 14.5 mm, and a 14.3- Fr single-lumen silicone catheter (Figure 1) for postoperative IP chemotherapy. The subcutaneous space was dissected and a pocket for implantation of the port was created in the right lower abdomen. A catheter was then inserted from the subcutaneous pocket into the peritoneal cavity, penetrating the abdominal wall, and the end of the catheter was placed in the pelvis.

The postoperative course was uneventful. On postoperative day 14 the patient began chemotherapy with S-1 (TS-1®; Taiho Pharmaceutical Company, Tokyo, Japan) at 80 mg/m^2^/day (2 weeks on, 2 weeks off) and docetaxel administered intraperitoneally at 45 mg/m^2^ (days 1 and 15). He was discharged on postoperative day 29 and underwent outpatient chemotherapy.

**Figure 2:**
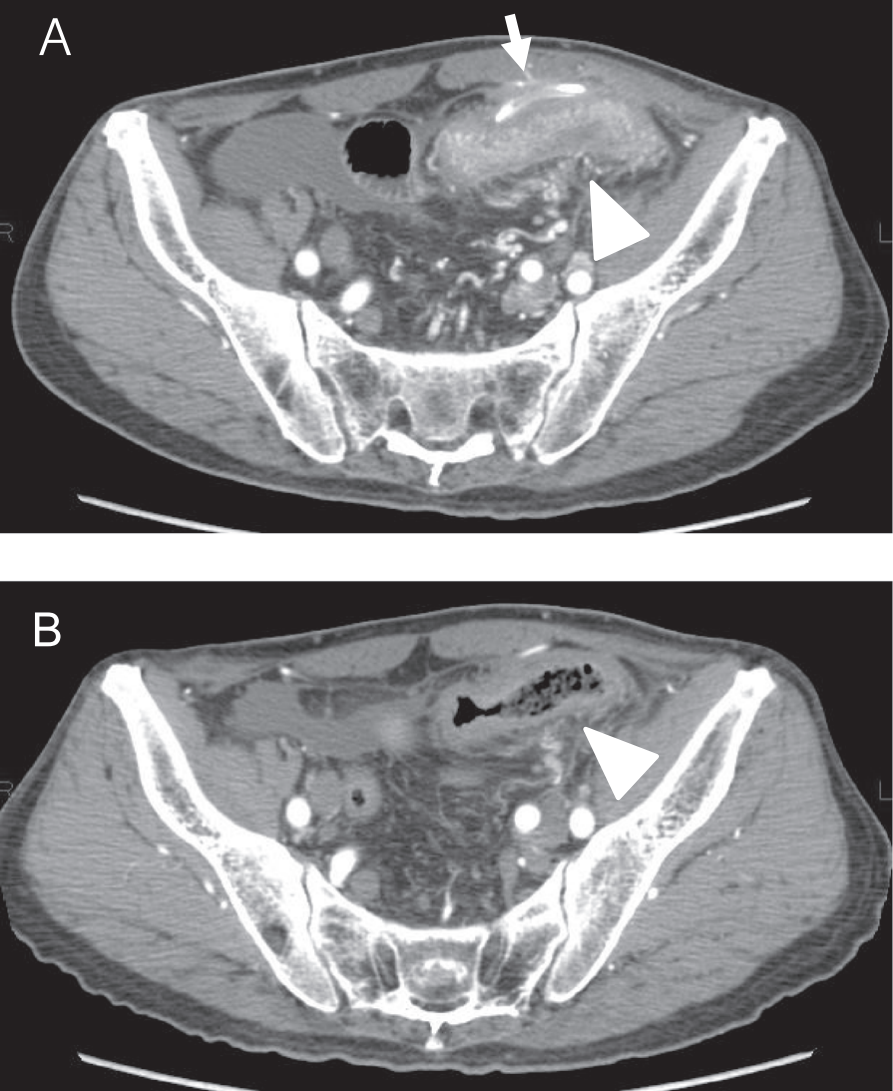
**Computed tomographic changes in sigmoid colon before and after removal of intraperitoneal access port system. (A)** A computed tomography scan showed marked wall thickening of the sigmoid colon (closed triangle) adjacent to the catheter (arrowhead) before removal. **(B)** Seven days after port removal, the wall thickening of the sigmoid colon had improved (open triangle).

**Figure 3:**
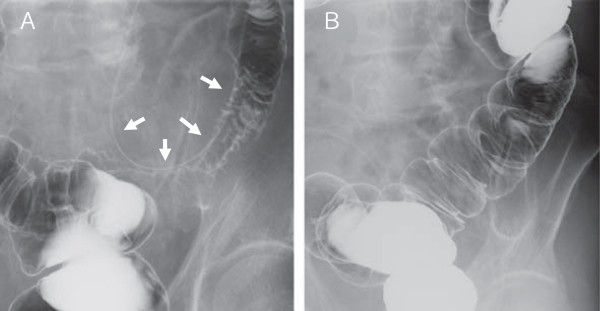
**Extrinsic irregular stenosis of the sigmoid colon on barium enema. (A)** Barium enema showed extrinsic irregular stenosis of the sigmoid colon around the catheter (arrowheads). **(B)** Four months after removal of the port system, the stenosis of the sigmoid colon had completely disappeared.

Two months after implantation of the IP access port the patient developed vomiting and abdominal pain. An abdominal X-ray showed dilatation of the small intestine. He was alert upon admission to department of gastroenterologic surgery of our hospital, with a blood pressure of 110/53 mmHg, a pulse rate of 64 beats/min, and a body temperature of 36.8°C. A physical examination was performed and localized tenderness was seen in the left lower abdomen apart from the port site. Neither port-site erythema nor swelling was observed. Laboratory data showed normal leukocyte and neutrophil counts (8420/μL and 4910/μL, respectively) and a slightly elevated C-reactive protein level (3.6 mg/dL). He was admitted with a diagnosis of ileus and a nasogastric tube was inserted. However, the left lower abdominal pain persisted.Anabdominal computed tomography scan revealed marked wall thickening and narrowing of the lumen of the sigmoid colon adjacent to the catheter of the IP access port system (Figure 2A). A barium enema also demonstrated extrinsic irregular stenosis of the sigmoid colon (Figure 3A). We initially suspected peritoneal metastasis from gastric cancer and considered colostomy as a palliative operation. However, the stenosis was determined to have resulted from inflammation due to catheter infection because the intestinal stenosis was confined to the sigmoid colon, which was not a common site of peritoneal metastasis such as transverse-colon or rectum, and the disease progression was too fast compared with the usual course of peritoneal metastasis. Therefore, we removed the IP access port system under local anesthesia for a therapeutic diagnosis and began cephalosporin administration. At the time of port system removal, there was no abscess in the subcutaneous space and no fibrin clots were occluding the lumen of the catheter.However, after the port system removal the patient’s abdominal pain immediately disappeared and he could ingest food normally several days later. In addition, an abdominal computed tomography scan demonstrated improvement in the thickening of the sigmoid colon wall seven days after port system removal (Figure 2B). Coagulase-negative staphylococci were detected by catheter culture. A colonofiberscopy showed no diverticula in the sigmoid colon, and that the mucosa was intact. Based on the patient’s clinical course and the above-described findings, we conclusively attributed the colonic stenosis to infection of the catheter. He was discharged on the 21 days after admission. The stenosis of the sigmoid colon had completely disappeared four months after port system removal as shown by a barium enema (Figure 3B).

Outpatient chemotherapy with S-1 and intravenous docetaxel was performed. A re-laparoscopy was subsequently performed to evaluate the patient’s response to chemotherapy. There were abnormal findings such as fibrosis, scar formation, or macroscopic progression of peritoneal metastasis in the sigmoid colon and its mesentery.

An IP access port system was re-implanted four months after removal. The patient remains alive without progression of peritoneal metastasis six months after surgery.

## Discussion

A well-established principle of IP chemotherapy is the regional pharmacologic advantage achieved by direct instillation of drugs into the peritoneal cavity. A recent phase II study of intravenous and IP paclitaxel combined with S-1 showed a 1-year overall survival rate of 78% and a median survival time of 22.5 months for patients with peritoneal metastasis from gastric cancer [6]. Other clinical trials involving IP chemotherapy with taxane agents have also shown favorable prognoses, with a median survival time of 16.2 to 24.6 months [7-9]. A multicenter randomized clinical trial is now ongoing to generate evidence regarding the effects of IP chemotherapy on gastric cancer with peritoneal metastasis.

To date, several reports have investigated complications associated with implanted subcutaneous ports and catheters for IP treatment of ovarian and gastric cancer [10-19]. Infection and malfunction were the most common complications; the rate of infection was reportedly 2.1% to 10.7%, and the rate of malfunction was 3.7% to 18.2% (Table 1). Emoto *et al.* reported that intestinal bacteria were found in bacterial cultures of lavage fluid obtained through the port in six out of nine patients [19]. Although a relationship appears to exist between infection and gastrointestinal surgery performed concurrently with port implantation, no evidence of this relationship has been found in previous retrospective studies. In our patient, we implanted subcutaneous ports and catheters concurrently with total gastrectomy, but the catheter culture revealed coagulase-negative staphylococci. This suggests that the catheter infection was due to contamination of the port site at the time of needling or contamination via the infusion device because coagulase-negative staphylococci are the resident bacteria of the skin. It is important for medical staff members to take standard precautions to prevent port infections as presented here.

**Table 1:** Complications associated with intraperitoneal access port systems in previous reports *n: number of patient

**Author**	**Year**	*n	**Type of cancer**	**Malfunction**	**Infection**	**Bowel perforation/fistula**	**Small bowel obstruction**	**Total**
				*n (%)	*n (%)	*n (%)	*n (%)	*n (%)
Pfeifle *et al.*[18]	1984	54	ovarian cancer	3 (5.5)	3 (5.5)	0	0	6(11)
Piccart *et al.*[12]	1985	145	ovarian cancer	3 (2.1)	12 (8.3)	2 (1.4)	0	17 (11.7)
Braly *et al.*[17]	1986	33	ovarian cancer	2 (6.1)	6 (18.2)	1 (3.0)	1 (3.0)	10 (30.3)
Davidson *et al.*[13]	1991	227	ovarian cancer	20 (8.8)	12 (5.3)	8 (3.5)	0	40 (17.6)
Malmastorm *et al.*[14]	1994	125	ovarian cancer	6 (4.8)	5 (4.0)	0	0	37 (30)
Topuz *et al.*[10]	2000	56	ovarian cancer	6 (10.7)	3 (5.4)	1 (1.8)	0	10 (18)
Makhija *et al.*[15]	2001	301	ovarian cancer	19 (6.3)	11 (3.7)	0	0	30 (10)
Walker *et al.*[16]	2006	205	ovarian cancer	18 (8.8)	21 (10)	4 (2.0)	0	40 (20)
Emoto *et al.*[19]	2012	131	gastric cancer	10 (7.6)	9 (6.9)	2 (1.5)	0	27 (21)

Previous studies have reported that gut-associated complications included fistula formation and perforation (0.0 to 3.5%). Braly *et al.* also reported one case of small bowel obstruction due to an IP catheter [17]. Fibrous sheath formation around the catheter was thought to pose a risk of small bowel obstruction [20].

To the best of our knowledge this is the first case of colonic stenosis as a complication of an IP port. We could not clarify the mechanism of the stenosis of the sigmoid colon in this case. We speculate that inflammation secondary to the catheter infection was the main contributor to the wall thickening and subsequent stenosis of the sigmoid colon because we did not observe numerous fibrous sheaths attached to the catheter at the time of removal. An important point of our case is that the extrinsic stenosis was remarkably similar in appearance to peritoneal metastasis. In general, peritoneal metastasis from gastric cancer frequently involves stenosis of the colorectum. In this case, the gut stenosis was not histologically confirmed by colonofiberscopy because it extended from the serosal side of the colonic wall. The findings obtained by barium enema were characteristic of the metastatic carcinoma that extended from the serosal side of the colonic wall.

Our patient had no fever and no severe acute inflammatory changes were found upon blood examination during the infectious episode. Therefore, it was difficult to distinguish whether infection or peritoneal metastasis caused the colonic stenosis. Such a diagnosis should be cautiously obtained because it substantially influences the subsequent treatment plan.

The occurrence of port complications in gastric cancer has been investigated in only one other report [19] according to our review of the literature, and has not been fully eliminated as is the case in ovarian cancer. Because peritoneal metastasis of gastric cancer more frequently involves enterostenosis than peritoneal metastasis of ovarian cancer, our case is thought to be significant in terms of the management of IP chemotherapy-induced complications, especially in patients with gastric cancer.

## Conclusions

In summary, we report the case of a rare complication in association with an IP access port system. Infection of the port system should be considered as a differential diagnosis when colonic stenosis adjacent to the catheter is observed during IP chemotherapy.

## Consent

Written informed consent was obtained from the patient for publication of this case report and accompanying images. A copy of the written consent is available for review by the Editor-in-Chief of this journal.

## Abbreviation

IP: intraperitoneal.

## Competing interests

The authors declare that they have no competing interests.

## Authors’ contributions

JK wrote the case report. SF and TF interpreted the data related to the oncologic disease. JK, TT, TW, KoO, KN, and KaO performed the physical examination and medical care. IM, HH, HN, TM, H Tajima, H Takamura, IN, HK, and TO contributed to the writing and revision of the manuscript. All authors read and approved the final manuscript.
